# Left atrial appendage closure in atrial fibrillation patients with prior major bleeding or ineligible for oral anticoagulation

**DOI:** 10.1007/s12471-019-1295-5

**Published:** 2019-06-11

**Authors:** L. I. S. Wintgens, V. M. M. Vorselaars, M. N Klaver, M. J. Swaans, A. Alipour, B. J. W. M. Rensing, M. C. Post, L. V. A. Boersma

**Affiliations:** 1 Department of Cardiology, St. Antonius Hospital, Nieuwegein, The Netherlands; 2 Department of Cardiology, Rivierenland Hospital, Tiel, The Netherlands; 3 Department of Cardiology, AMC Amsterdam, Amsterdam, The Netherlands

**Keywords:** Atrial fibrillation, Left atrial appendage closure, Catheter ablation, Stroke prevention, Bleeding

## Abstract

**Aims:**

Oral anticoagulation (OAC) reduces the ischaemic stroke risk in patients with atrial fibrillation (AF), but in turn leads to an increased risk of adverse bleeding events. Alternatively, left atrial appendage closure (LAAC) using a mechanical device might overcome these bleeding complications. However, evidence regarding LAAC in patients at high bleeding risk is scarce. This study evaluates the clinical features of AF patients with previous bleeding that underwent LAAC.

**Methods:**

In this retrospective cohort study patients with previous major bleeding or a bleeding predisposition scheduled for transcatheter LAAC were included. The frequency and type of previous bleeding events and prevalence of bleeding and ischaemic stroke during follow-up were evaluated.

**Results:**

A total of 73 patients (58% male, age 72.1 ± 7.2 years; CHA_2_DS_2_-VASc 4.5 [3.0–5.0]; HAS-BLED 4.0 [3.0–4.0]; 46% paroxysmal AF) were included. Previous bleeding occurred from intracranial (*n* = 50, 69%), gastro-intestinal (*n* = 13, 18%) or multiple (*n* = 16, 22%) foci. After OAC discontinuation due to bleeding, 19% suffered subsequent stroke. LAAC was successful in 96% of patients. During a median of almost 3 years’ follow-up recurrent major bleeding occurred in 4 patients (5.5%) despite OAC discontinuation in 93.2%. A total of 6 ischaemic strokes were observed, resulting in an annualised stroke rate of 2.9% compared to a calculated expected stroke rate of 6.7%.

**Conclusions:**

Percutaneous LAAC may provide an alternative strategy to long-term OAC therapy in AF patients with a high bleeding risk. During follow-up, both ischaemic stroke and recurrent bleeding rates were lower than expected based on the CHA_2_DS_2_-VASc and HAS-BLED scores respectively.

## What’s new?

- Left atrial appendage closure (LAAC) provides an alternative to long-term oral anticoagulation (OAC) for stroke prevention in atrial fibrillation (AF) patients.
- LAAC may reduce stroke risk and eliminate the need for OAC therapy in patients ineligible for OAC.
- We present one of the larger registries of patients with previous major bleeding or very high bleeding risk that underwent transcatheter LAAC.
- During long-term follow-up, stroke and bleeding rates were lower than expected based on CHA_2_DS_2_-VASc and HAS-BLED scores respectively, despite discontinuation of OAC in 93.2% of patients.
- Percutaneous LAAC may provide an alternative strategy to long-term OAC therapy in AF patients with a very high bleeding risk or a history of major bleeding.

## Introduction

Thromboembolic clots formed in the left atrial appendage account for up to 90% of atrial fibrillation (AF)-related strokes in patients with AF [1]. Although oral anticoagulation (OAC) therapy with vitamin K antagonists (VKA) significantly reduces the risk of stroke, this is associated with an increased risk of adverse (major) bleeding events. Even the non-VKA OACs (NOACs) are associated with an increased bleeding risk [2–6]. Moreover, patients with a history of major bleeding or contra-indications for OAC have always been excluded from the randomised trials.

Stroke prevention strategies are particularly challenging in patients in whom (N)OAC is contra-indicated. The need for OAC should be based on a careful individualised assessment of both stroke and bleeding risk, indicated as CHA_2_DS_2_-VASc and HAS-BLED score respectively [7]. Left atrial appendage closure (LAAC) using a mechanical device can provide an alternative to lifelong anticoagulation. Two large-scale randomised controlled trials have demonstrated the safety and efficacy of the Watchman device compared to long-term OAC therapy for stroke prevention [8, 9]. However, in these trials only patients without contra-indications for long-term OAC were included. Evidence for LAAC in patients with a history of major bleeding under (N)OAC is scarce, yet this is expected in the designated patient population in whom this therapy may be considered in current ESC guidelines [10]. In this observational cohort study, we report the clinical features of a real-world series of consecutive patients with previous major bleeding or very high bleeding risk that underwent transcatheter LAAC in our centre. The aim of the study was to show the procedural efficacy and safety and long-term outcome data in this selected population of AF patients that seeks an alternative to OAC because of severe bleeding problems in the past, and for which there is so far very limited evidence in the literature.

## Methods

In this retrospective single-centre cohort study, consecutive patients scheduled for percutaneous LAAC, with non-valvular AF and contra-indications to OAC therapy, including previous major bleeding and very high tendency to fall, were included. Patients who underwent stand-alone LAAC as well as patients scheduled for a combined procedure with catheter ablation (CA) followed by LAAC were included.

### Data collection

Data on percutaneous LAAC are collected prospectively by means of a web-based database. We recorded the rate and type of previous bleeding events, management of anticoagulation after bleeding, peri-procedural characteristics and prevalence of major bleeding and ischaemic stroke during follow-up.

### Patient and procedural management

All patients visited a cardiologist-electrophysiologist for evaluation of eligibility for percutaneous LAAC. Patients with an indication for LAAC were evaluated in multidisciplinary team meetings.

Transoesophageal echocardiography (TOE) was performed in all patients prior to the procedure to evaluate the LAA anatomy and to exclude intracardiac thrombus.

LAAC could be performed by either of the available percutaneous techniques used in our centre, Watchman (Boston Scientific, Natick, MA, USA) or Amplatzer Amulet (Abbott, Minneapolis, MN, USA). The choice of technique was left to the physician’s discretion.

If patients were scheduled for the combined procedure, CA was performed prior to LAAC. All patients underwent post-procedural chest radiography to confirm the proper position of the device.

### Follow-up

All patients were seen by their treating cardiologist-electrophysiologist in the outpatient clinic 3 and 12 months after the procedure. In addition, regular telephone interviews were performed.

Rhythm monitoring was performed through electrocardiograms (ECGs) and Holter recordings at 6 and 12 months of follow-up. Patients with symptoms suggestive of recurrent atrial arrhythmias were encouraged to obtain symptom-driven ECG recordings.

### Antithrombotic therapy

The recommended post-implant regimen consisted of (1) clopidogrel and aspirin for 1–3 months (for Amplatzer devices) and for 6 months (for Watchman devices) post-implantation, (2) aspirin indefinitely.

However, the choice and duration of post-implant antithrombotic medication was not mandatory and was left to the physician.

TOE was repeated between 45 and 60 days post-procedure to evaluate device position, residual flow and thrombus formation.

### Outcome

In this observational cohort study, we report on the incidence of ischaemic stroke during clinical follow-up. In the absence of a control group, the observed stroke rate was compared to the predicted stroke rate based on the CHA_2_DS_2_-VASc score [11].

Secondary outcome included procedural success, peri-procedural complications up to 30 days, management of antithrombotic medication, freedom from atrial tachyarrhythmias and major bleeding during follow-up.

Major bleeding was defined as bleeding type 3 or greater according to the BARC criteria [12].

## Results

### Patient selection and characteristics

Between 2010 and 2016, a total of 135 patients were scheduled for percutaneous LAAC in our institution, of whom 73 were included in this study. Patient characteristics at baseline are shown in Tab. 1.

**Table 1:** Baseline and peri-procedural characteristics

*Total no. of patients*	73
*Age, years* *±SD*	72.1 ± 7.2
*Male*	42 (58%)
*Paroxysmal AF*	32 (44%)
*Persistent or long-standing persistent AF*	41 (56%)
*CHADS*_*2*_	3.0 [2.0–4.0]
*CHA*_*2*_*DS*_*2*_*-VASc*	4.5 [3.0–5.0]
*HAS-BLED*	4.0 [3.0–4.0]
*History of stroke*	30 (41%)
*History of bleeding*	70 (96%)
*Intracranial bleeding*	50 (69%)
*Gastro-intestinal bleeding*	13 (18%)
*Pulmonary bleeding*	2 (3%)
*Other bleeding site*	17 (33%)
*Multiple bleeding sites*	16 (22%)
*Indefinite OAC withdrawal after bleeding*	63 (86%)
*Stroke after OAC withdrawal*	14 (19%)
*Stand-alone LAAC procedure*	45 (61.6)
*Combined CA and LAAC*	28 (38.4)
*Device type*	
Watchman	69 (94.5)
Amplatzer Amulet	4 (5.5)
*Successful LAAC*	70 (95.9)
Complete LAA closure	67 (95.7)
Minimal residual flow	3 (4.3)
*Number of devices used*	1.0 [1.0–1.0]
*Total procedure time, min*	92 ± 34
*Total procedure time stand-alone, min*	78 ± 31
*Fluoroscopy time, min*	11 ± 5
*Major 30-day complications*	6 (8.2)
Pericardial effusion	0 (0.0)
Intracoronary air embolus	1 (1.4)
Device embolisation	2 (2.7)
Stroke	1 (1.4)
TIA	2 (2.7)
Death	0 (0.0)
*Minor 30-day complications*	3 (4.1)
Groin haematoma	3 (4.1)

*AF* atrial fibrillation, *CA* catheter ablation, *LAA* left atrial appendage, *LAAC* left atrial appendage closure, *OAC* oral anticoagulation, *TIA* transient ischaemic attack

### Previous bleeding, stroke, and antithrombotic management

Details of the prior bleeding events are shown in Tab. 1. Seventy patients (96%) had a history of previous major bleeding. Three patients (4%) were considered ineligible for OAC due to severe thrombocytopenia or elevated falling risk due to narcolepsy and post-dystrophic muscular dystrophy. In the majority of patients (86%) oral antithrombotic therapy had been discontinued or never started. Fourteen patients (19%) suffered a stroke after OAC withdrawal.

### Procedural characteristics

Procedural characteristics are shown in Tab. 1. LAAC was performed as a stand-alone procedure in 45 of 73 patients (62%). In 28 patients LAAC was combined with CA.

Implantation of the LAAC device was unsuccessful in 3 patients: one device dislocation to the left ventricular outflow tract during the procedure and 2 patients with unsuitable anatomy. In 70 patients (95.9%) an LAAC device could be implanted, leading to successful LAAC in all 70.

Serious peri-procedural complications up to 30 days occurred in 6 (8.2%) patients (2 patients with a combined procedure, 4 patients with a stand-alone LAAC procedure).

Peri-procedural dislocation of the Watchman device occurred in 2 patients (2.7%); in one patient a new device could be successfully implanted in the LAA. Two transient ischaemic attacks (TIAs) and one ischaemic stroke were reported to occur within 24 h of the procedure. TOE was performed in all these patients, showing complete closure of the LAA with no residual flow and no device thrombus. In the patient with a stroke, left atrial thrombus had been seen on TOE after the ablation sheath was exchanged for the Watchman sheath. Despite an extra 5,000 IU of heparin, this patient suffered an ischaemic stroke hours after the procedure with residual complaints. No peri-procedural major bleeding, pericardial effusion, tamponade or death occurred. Minor groin haematoma was seen in 3 patients (4.1%).

### Transoesophageal echocardiographic follow-up

TOE was performed within 45–60 days in 69 of 70 (98.6%) patients with a successfully implanted LAAC device, showing successful sealing of the LAA in 68 of 69 (98.5%) and significant residual flow in 1 of 69. No device thrombi were found.

### Antithrombotic therapy management

Table 2 shows pre- and post-implant oral antithrombotic therapy in all 73 patients.

**Table 2:** Antithrombotic therapy management

	Pre-procedural	3 months	End of FU
*VKA*	8 (11.0%)	5 (6.8%)	2 (2.7%)
*NOAC*	1 (1.4%)	1 (1.4%)	3 (4.1%)
*DAPT*	0 (0.0%)	16 (21.9%)	6 (8.2%)
*SAPT*	36 (49.3%)	45 (61.6%)	52 (71.2%)
*None*	28 (38.3%)	6 (8.2%)	10 (13.7%)

*VKA* vitamin K antagonist, *NOAC* non-vitamin K antagonist oral anticoagulation, *DAPT* dual antiplatelet therapy, *SAPT* single antiplatelet therapy, *FU* follow-up

After 35.5 months of follow-up, only 5 patients were still receiving VKA (2) or NOAC (3) therapy. Fifty-eight patients were on either single (71%) or dual (8%) antiplatelet therapy, while 10 patients (14%) were receiving no antithrombotic medication at all.

### Clinical outcome

Table 3 shows the clinical outcome of the patients after a median follow-up period of 35.5 months.

**Table 3:** Clinical outcome after 35.5 months of follow-up

*No. of patients*	73
*Minor bleeding*	2 (2.7)
Epistaxis	1 (1.4)
Subcutaneous haematoma	1 (1.4)
*Major bleeding*	4 (5.5)
Intracranial	1 (1.4)
Gastro-intestinal	2 (2.7)
Pulmonary	1 (1.4)
*Annualised bleeding rate*	1.8%
*Overall ischaemic stroke*	6 (8.2)
*Ischaemic stroke in patients with successfully implanted LAAC device*	6 (8.5)
*Ischaemic stroke in patients with successfully implanted LAAC device and cessation of (N)OAC*	6 (9.0)
*Annualised stroke rate*	3.0

#### Thromboembolic events

One peri-procedural stroke (1.4%) was observed within 30 days of LAAC implantation. From 30 days until 35.5 months of follow-up, a total of 5 ischaemic strokes (6.8%) were recorded. The overall stroke rate was 6 of 73 included patients and 6 of 70 patients after successful LAAC implantation. This results in an annualised stroke rate of 2.9%. The annualised stroke risk of 2.9% was compared to an estimated stroke rate of 6.7% based on CHA_2_DS_2_-VASc score [11], accounting for a 57% risk reduction.

In 3 of 5 patients TOE data after TIA or stroke were available, showing complete closure in 2 patients and minimal residual flow of 2 mm in 1, and no device thrombus. Notably, 4 patients continued NOAC or OAC therapy owing to repeated electrical cardioversions and were thus at lower stroke risk. When these patients were excluded from the stroke risk analysis, the annualised stroke risk is 3.0%.

All patients were on antiplatelet therapy at the time of stroke (5 on single antiplatelet therapy, 1 on dual antiplatelet therapy).

#### Bleeding events

Three patients (4.1%) had four major bleeding events (5.5%) from 30 days until 35.5 months of follow-up, resulting in an annualised bleeding event rate of 1.8%. Bleeding occurred at the following foci: intracranial (*n* = 1), gastro-intestinal (*n* = 2) and pulmonary (*n* = 1). There was a relative risk reduction of 80% between the observed major bleeding rate of 1.8% and the expected bleeding rate of 8.9% under (N)OAC use. No fatal bleeding events occurred during follow-up.

#### Atrial fibrillation recurrence

Recurrent AF was seen in 19 of 28 patients that underwent combined CA and LAAC (67.9%).

## Discussion

This study reports the clinical features of a real-world series of patients with a very high bleeding risk that underwent transcatheter LAAC in our centre. In our population previous bleeding most often occurred (69%) from intracranial foci, while after OAC discontinuation to avoid bleeding 19% of patients suffered from ischaemic stroke. LAAC was found to be feasible in this very vulnerable patient cohort, with stroke and bleeding rates during 3 years of follow-up much lower than expected based on CHA_2_DS_2_-VASc and HAS-BLED scores respectively.

The long-term benefit of stroke and bleeding reduction by LAAC therefore appears to outweigh the procedural risks and seems preferable to both avoidance and continuation of OAC.

Current guidelines state that LAAC may be considered in AF patients with contra-indications for long-term OAC therapy [10]. However, in the randomised PROTECT-AF and PREVAIL trials, which demonstrated the safety and efficacy of Watchman LAAC, patients with prior bleeding were not included [8, 9]. Even in recent large real-world registries of LAAC less than half of the patients had an actual history of prior bleeding, and no sub-analysis has been performed for this population thus far [13, 14].

The only data focussing on this particular population come from smaller registries and are actually scarce [5]. Several small “real-world” cohort studies using various percutaneous devices [16–20] have indicated that LAAC appears to be feasible and safe in patients with a history of major bleeding of intracranial or gastro-intestinal origin. Our present study with 96% of patients with a history of major bleeding is in line with those data and represents a larger cohort of patients that were not selected according to type of bleeding.

### The therapeutic dilemma in contra-indicated patients

The HAS-BLED score is widely used to estimate the risk of major bleeding in patients with AF. However, it entails important drawbacks, including limited sensitivity and specificity and the fact that it does not account for type of bleeding or recurrence rate. Patients who have suffered from multiple bleeding foci and/or multiple bleeding events, as 22% of patients in our cohort did, clearly have a much higher risk of future recurrent bleeding, which is not reflected as an increase in their HAS-BLED score.

Furthermore, as CHADS-VASc and HAS-BLED scores share several risk factors, individual bleeding and stroke risk increase in parallel, creating a therapeutic dilemma in patients with very high risk of both stroke and bleeding.

Such patients are often forced to discontinue all antithrombotic medication, leaving them with a substantially increased stroke risk. Indeed, our study found that 19% of patients suffered from ischaemic stroke after OAC was withdrawn owing to previous bleeding. A strategy of LAAC with an annualised stroke rate of only 2.9% therefore appears to be more effective than providing these patients with no alternative to anticoagulation.

Previous studies have reported major bleeding in the peri-procedural period ranging from 1.0% to 5.1% [8, 13, 21].

In our cohort of patients with a history of major bleeding, we did not observe a single peri-procedural major bleeding event. Only minor groin haematomas that could be managed conservatively were observed in 4.1%. The operators were experienced in cardiac catheterisation and left atrial procedures, and these contra-indicated patients in general had lower peri-procedural anticoagulation use. However, an overall complication rate of 8.2% was found, which is in line with the PROTECT-AF and ASAP registries but slightly higher than in the PREVAIL and EWOLUTION registries [8, 13, 15, 21]. The less aggressive antithrombotic strategies in the peri-procedural phase may have contributed to the peri-procedural thromboembolism rate.

In our series the actual annualised stroke risk of 2.9% compared favourably to an estimated stroke risk of 6.7% based on CHA_2_DS_2_-VASc score, constituting a 57% risk reduction.

This is in line with the 62% ischaemic stroke risk reduction observed in the PREVAIL registry, and only slightly lower than the ischaemic stroke risk reductions in the PROTECT-AF, CAP and EWOLUTION registries [8, 22, 23].

### Limitations

This study is subject to the limitations associated with a retrospective study design, including the lack of a control group and a relatively small sample size.

We emphasise that our study was inevitably subject to selection bias, including only patients that were referred for LAAC. AF-related ischaemic strokes as well as OAC-related bleeding events have been associated with high morbidity and mortality [24]. Naturally, this patient cohort consisted only of patients who survived a major bleeding event and were eligible to undergo LAAC. Patients who subsequently died or suffered from severe (neurological) deficit were probably not referred to our centre. Therefore, there is a probability that the magnitude of this problem could have been larger than reported in our study.

Furthermore, the study cohort consisted of a heterogeneous patient group at vastly differing risks for (recurrent) bleeding and stroke. Consequently, different LAAC devices were used and various pre- and post-implant antithrombotic strategies were applied.

Future larger prospective and randomised controlled trials are needed to provide more knowledge on LAAC in this challenging subgroup of patients. They are among patients with the highest stroke and bleeding risk and are therefore expected to benefit most from this procedure.

## Conclusion

Percutaneous LAAC may provide an alternative strategy to long-term OAC therapy in AF patients with a very high bleeding risk due to previous major bleeding. During follow-up, both ischaemic stroke and recurrent bleeding rates were lower than expected based on the CHA_2_DS_2_-VASc and HAS-BLED scores respectively.
